# Inflammasomes as therapeutic targets in human diseases

**DOI:** 10.1038/s41392-021-00650-z

**Published:** 2021-07-02

**Authors:** Yangxin Li, Hui Huang, Bin Liu, Yu Zhang, Xiangbin Pan, Xi-Yong Yu, Zhenya Shen, Yao-Hua Song

**Affiliations:** 1 Institute for Cardiovascular Science and Department of Cardiovascular Surgery, First Affiliated Hospital and Medical College of Soochow University, Collaborative Innovation Center of Hematology, Soochow University, Suzhou, Jiangsu P. R. China; 2 Cardiovascular Department, The Eighth Affiliated Hospital, Sun Yat-sen University, Shenzhen, Guangdong P. R. China; 3 Department of Cardiology, The Second Hospital of Jilin University, Changchun, Jilin P. R. China; 4 Department of Cardiac Surgery, Fuwai Hospital, Beijing, P. R. China; 5 Key Laboratory of Molecular Target & Clinical Pharmacology and the State Key Laboratory of Respiratory Disease, Guangzhou Medical University, Guangzhou, Guangdong P. R. China; 6 Cyrus Tang Hematology Center, Collaborative Innovation Center of Hematology, Soochow University, National Clinical Research Center for Hematologic Diseases, the First Affiliated Hospital of Soochow University, State Key Laboratory of Radiation Medicine and Protection, Soochow University, Suzhou, China

**Keywords:** Inflammation, Preclinical research

## Abstract

Inflammasomes are protein complexes of the innate immune system that initiate inflammation in response to either exogenous pathogens or endogenous danger signals. Inflammasome multiprotein complexes are composed of three parts: a sensor protein, an adaptor, and pro-caspase-1. Activation of the inflammasome leads to the activation of caspase-1, which cleaves pro-inflammatory cytokines such as IL-1β and IL-18, leading to pyroptosis. Effectors of the inflammasome not only provide protection against infectious pathogens, but also mediate control over sterile insults. Aberrant inflammasome signaling has been implicated in the development of cardiovascular and metabolic diseases, cancer, and neurodegenerative disorders. Here, we review the role of the inflammasome as a double-edged sword in various diseases, and the outcomes can be either good or bad depending on the disease, as well as the genetic background. We highlight inflammasome memory and the two-shot activation process. We also propose the M- and N-type inflammation model, and discuss how the inflammasome pathway may be targeted for the development of novel therapy.

## Introduction

Inflammasomes are intracellular multimeric complex molecules that recognize either pathogen-associated molecular patterns (PAMPs) or danger-associated molecular patterns (DAMPs).^1,2^ The nucleotide-binding oligomerization (NOD)-, leucine-rich repeat (LRR)-, and pyrin domain-containing 3 (NLRP3) and the NOD-, lRR-, and CARD-containing 4 (NLRC4) inflammasomes belong to the NOD-like receptor (NLR) family.^3,4^ Non-NLR proteins, such as absent in melanoma 2 (AIM2), form inflammasomes that can sense cytosolic double-stranded DNA.^5,6^

**Fig. 1:**
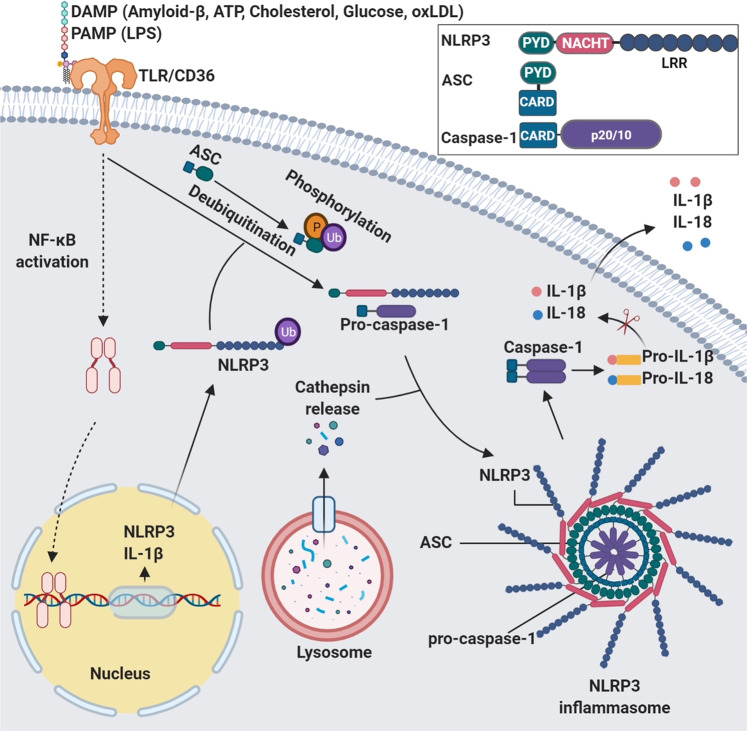
Mechanisms of NLRP3 inflammasome assembly and activation. NLRP3 needs to be primed prior to activation. The priming process includes the binding of PAMPs/DAMPs to TLR or CD36, which promotes the transcription of NLRP3 and IL-1β. The priming event also includes de-ubiquitination of NLRP3. The ubiquitination and phosphorylation of the adaptor protein ASC is also necessary for the assembly of inflammasome. Disruption of the lysosome and release of cathepsin enhances the activation of NLRP3 inflammasome. The assembly of activated inflammasome leads to the processing of pro-caspase-1 into mature and active enzyme, which in turn cleaves pro-IL-1β and pro-IL-18 into active cytokines. Inset: activated NLRP3 binds to ASC through PYD–PYD interactions. Pro-caspase-1 binds to ASC through CARD–CARD interactions

**Fig. 2:**
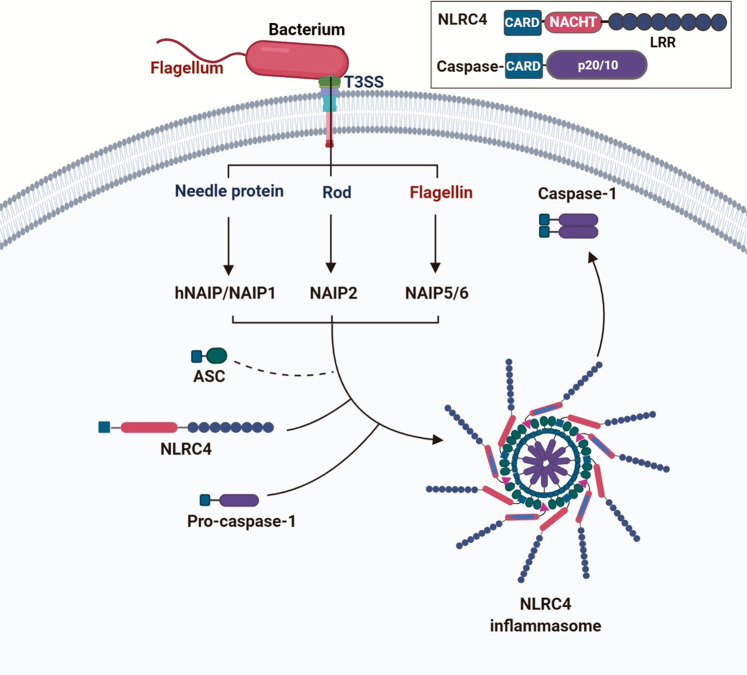
Mechanisms of NLRC4 inflammasome assembly and activation. The bacterial ligands such as needle protein, rod, and flagellin bind to NAIP proteins. Ligand-bound NAIP then interacts with NLRC4 to form the inflammasome through oligomerization. NLRC4 recruits pro-caspase-1 to the inflammasome via CARD–CARD interaction. Inset: NLRC4 inflammasome components

**Fig. 3:**
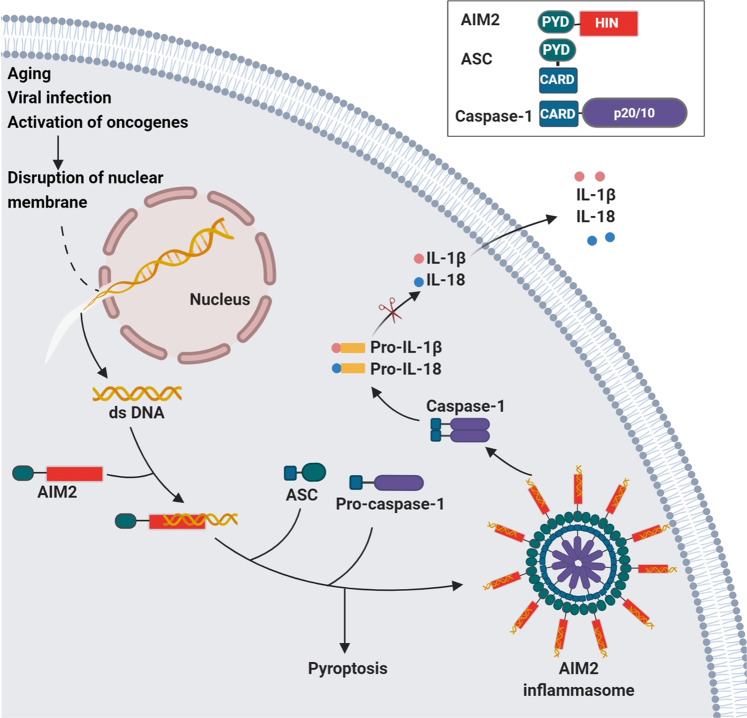
The formation and activation of AIM2 inflammasome. The HIN domain of AIM2 binds dsDNA in the cytosol. AIM2 binds to ASC through PYD–PYD interaction. The inflammasome is formed through AIM2-ASC oligomerization. Pro-caspase-1 is recruited by ASC via CARD–CARD interaction. Inset: AIM2 inflammasome components

**Fig. 4:**
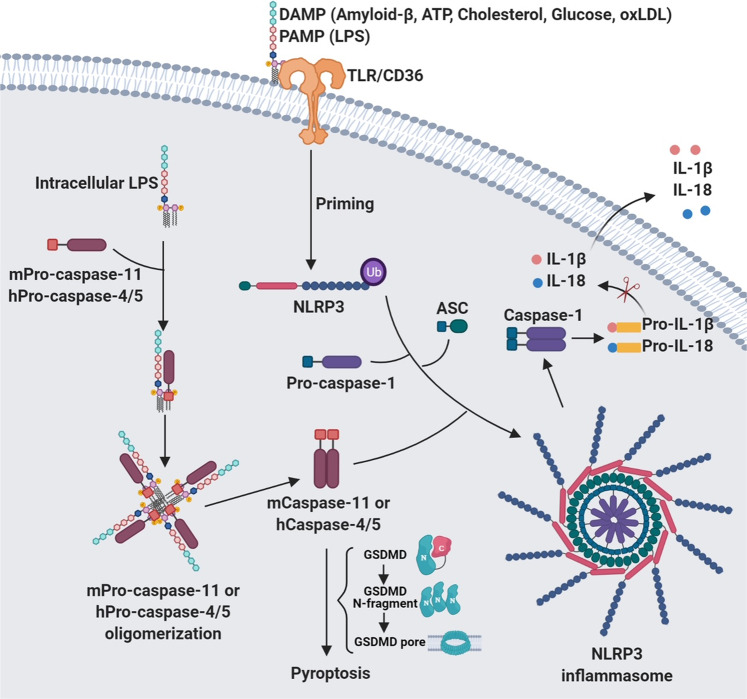
Mechanisms of non-canonical inflammasome assembly and activation. Intracellular LPS forms a complex with pro-caspase-11 (mouse) or pro-caspases-4 and -5 (human), which become activated through oligomerization. Activated caspase-4/5/11 induces pyroptosis by cleaving intact GSDMD to generate the N-terminal fragment which forms a pore on membrane. The active mCaspase-11/hCaspase-4 is also involved in the assembly and activation of the NLRP3 inflammasome when PAMP/DAMP signals are present

The canonical inflammasomes (NLRP3, NLRC4, and AIM2) serve as a platform to engage pro-caspase-1 (Figs. 1–3 and Box 1), which becomes active caspase-1 through the oligomerization of pro-caspase-1 proteins.^7^ Activated caspase-1 processes pro-interleukin-1β (IL-1β) and pro-IL-18 to generate their active forms, which induce pyroptosis, a pro-inflammatory form of cell death.^2,8–10^ The non-canonical inflammasomes activate caspase-11 (mouse) or caspase-4/5 (human) in response to Gram-negative bacteria-derived lipopolysaccharide (LPS) without cleaving pro-IL-1β (Fig. 4).^1,11,12^

NLRs have three functional domains: the amino-terminal domain includes a pyrin domain (PYD), or a caspase-recruitment domain (CARD); the central nucleotide-binding and oligomerization domain (NACHT) is present in all NLR proteins; and the carboxy-terminal domain is an LRR domain that binds to ligand.^13^ The PYD interacts with apoptosis-associated speck-like protein containing a CARD (ASC) which in turn binds pro-caspase-1 (Fig. 1).^14,15^ ASC is an adaptor protein used by many cytoplasmic pattern recognition receptors (PRRs) such as NLRs to recruit pro-caspase-1 to the inflammasomes via its CARD domain. PRRs are sensor proteins that detect signals to activate an inflammatory response.

The membrane-bound PRRs such as Toll-like receptors (TLRs) and cytosolic PRRs such as NLRs recognize PAMPs and host-derived DAMPs, respectively.^16^ Activation of PRRs by PAMPs leads to the production of type I interferons and chemokines, whereas activation of PRRs by DAMPs activates caspases that eventually lead to the production of pro-inflammatory cytokines. DAMP-induced inflammation is referred to as sterile inflammation because it occurs in the absence of invading microbes.^10,13,17^ DAMP-triggered sterile inflammation can exaggerate pathology either as a causative or contributing factor in response to host-derived factors such as intracellular molecules released from damaged cells. When the inflammation persists for a long period of time, it becomes a chronic process, leading to sterile inflammatory diseases such as atherosclerosis, myocardial infarction, diabetes, neurodegenerative disease, depression, and cancer.

Box 1 Glossary • *Apoptosis-associated speck-like protein containing a caspase-recruitment domain (ASC)*: an adaptor protein used by many cytoplasmic PRRs to engage with caspase-1 in an inflammasome complex.

• *Cathepsins*: A family of cysteine proteases located in lysosomes.

• *Danger-associated molecular patterns (DAMPs)*: direct ligands for PRRs that are generated by dead or dying cells, as well as inducers of sterile inflammatory diseases such as oxLDL, glucose, and amyloid.

• *Inflammasomes*: innate immune system receptors and sensors that induce the activation of caspase-1 and the formation of inflammatory cytokines in response to DAMPs or PAMPs. Non-canonical inflammasome: the caspase of the non-canonical inflammasome is caspase-11 (mouse) or caspase-4/5 (human) rather than the canonical inflammasome (caspase-1).

• *NLRP3 inflammasome priming*: a process that increases NLRP3 and pro-IL-1β expression to a level that is sufficient for oligomerization and activation of the inflammasome. NLRP3 inflammasome priming requires transcriptional upregulation of NLRP3 and pro-IL-1β expression, as well as post-translational modification of NLRP3. Priming is the first step and oligomerization is the second step toward inflammasome activation.

• *NLR members*: Members of the NLR family are classified based on their three structural domains. The N-terminal effector domain includes a pyrin domain (PYD), or a caspase-recruitment domain (CARD), or a baculovirus inhibitory repeat (BIR) domain. The central nucleotide-binding oligomerization domain (NACHT): is a feature common to all NLR proteins. The C-terminal domain is a leucine-rich repeat domain (LRR).

• *Pathogen-associated molecular patterns (PAMPs)*: direct ligands for PRRs that are of microbial origin.

• *Pattern recognition receptors (PRRs)*: receptors for ligands or signals that activate immune pathways.

• *Sterile inflammatory diseases*: DAMPs-triggered inflammation is termed sterile inflammation because it occurs in the absence of microbes. Sterile inflammatory diseases include atherosclerosis, type 2 diabetes, neurodegenerative disorders, cancer, etc.

## Discovery and mechanisms of activation of inflammasomes

Different types of inflammasome are briefly discussed in order to understand the role of their activation in disease states.

### History of inflammasomes

The term “inflammasome” was first coined in 2002 by Dr. Jurg Tschopp and colleagues.^2^ They described the inflammasome as a “caspase activating complex” consisting of caspase-1, caspase-5, Pycard (caspase interacting domain), and NALP1 (an old name for NLRP1), which was later referred to as the NLRP1 inflammasome and this complex is responsible for IL-1β maturation. Two years later, Dr. Tschopp’s research team also discovered NLRP3 inflammasome.^18^ Their study revealed increased activity of the NLRP3 inflammasome in macrophages isolated from patients with Muckle–Wells syndrome. The NLRP3 inflammasome became the best-characterized inflammasome because of its involvement in both innate and adaptive immunity. It is activated by either whole pathogens including fungi,^19^ bacteria,^20^ and viruses^21,22^ or by host-derived molecules such as fibrillar amyloid-β (Aβ) peptide,^23^ as well as extracellular ATP^24^ and glucose.^25^ Unlike NLRP1, NLRP3 does not harbor a CARD domain, but instead recruits pro-caspase-1 through ASC that functions as an adaptor protein connecting NLRP3 and caspase-1. NLRC4 (IPAF) gene was identified by Poyet et al.^26^ when searching for proteins that can activate pro-caspase-1. They cloned IPAF cDNA using a pair of PCR primers based on sequence similarity to the CARD of pro-caspase-1, and demonstrated that IPAF was able to activate pro-caspase-1 through CARD–CARD interaction. Due to its structural homology to other NLR proteins, IPAF was later renamed as NLRC4.

AIM2 inflammasome was discovered by several independent groups when searching for new DNA sensors using different approaches. Burckstummer et al.^5^ identified AIM2 using orthogonal proteomic and genomic screen for proteins that associate with DNA and are transcriptionally regulated by IFN-β. Roberts et al.^27^ analyzed cyotoplasmic extracts from bone marrow-derived macrophages for binding to double-stranded DNA (dsDNA) by electromobility shift assay and mass spectrometry and identified p202 as a repressor of caspase-1 and caspase-3. p202 is a member of HIN-200 family, which is a cluster of interferon-inducible genes including AIM2. Because AIM2 is the only family member that can form a heterodimer with p202, they investigated the effect of AIM2 silencing on caspase-1 and caspase-3 activation and found that knockdown of AIM2 completely inhibited the activation of the caspases, therefore confirming the role of AIM2 as an activator of caspase-1 and capase-3. Fernandes-Alnemri and colleagues searched the NCBI database for proteins with pyrin and oligonucleotide-binding domains, and identified four human proteins (IFI16, AIM2, IFIX, and MNDA) of the HIN-200 family that meet the criteria. Among these proteins, AIM2 was the only one that can activate caspase-1 when ectopically expressed in 293T-caspase-1-ASC cell line.^28^ They further showed that cytoplasmic DNA triggers formation of AIM2 inflammasome by inducing AIM2 oligomerization. Hornung et al.^29^ identified AIM2 by searching for proteins containing a PYD domain and DNA binding domain and demonstrated that AIM2 is a receptor for cytosolic dsDNA and activates caspase-1 by forming a complex with ASC. The NLRP1, NLRP3, NLRC4, and AIM2 inflammasomes were termed as “canonical inflammasomes” because all of these inflammasomes are capable of activating caspase-1. The “non-canonical” inflammasome was recently discovered by several research teams when searching sensors for intracelluar LPS and upstream activator of caspse-11.^12,30,31^ They showed that, rather than triggering the activation of caspase-1, the non-canonical inflammasome activate caspase-11 in mice and caspase-4 and caspase-5 in humans in response to LPS. Activated caspase-4/5/11 cleaves intact GSDMD to generate the N-terminal fragment which bind to cell membrane, oligomerize, and form pores to induce pyroptosis (Fig. 4).

### Canonical inflammasomes

The activation of NLRP3 inflammasome can be triggered by molecules associated with both PAMPs and DAMPs (Fig. 1).^9,32^ In most cell types, NLRP3 needs to be primed by pattern recognition molecules or other receptors such as scavenger receptor CD36 before it can be activated. Binding of LPS to TLR4 is considered a priming event.^13^ Nuclear factor-κB (NF-κB) signaling is involved in priming NLRP3 for activation.^33^ Recent findings have shown that signaling by the PRR primes NLRP3 by inducing its de-ubiquitination in a non-transcriptional fashion.^34,35^ Once primed, the NLRP3 inflammasome is assembled and activated. In addition, linear ASC ubiquitination is required for NLRP3 inflammasome assembly.^36^ NLRP3 inflammasome formation can be induced by the products of damaged cells such as ATP, crystalline substances, nucleic acids, or by pathogen-derived molecules from infection.^1,3^ Of note, molecules formed under pathological conditions such as high glucose, oxidized low-density lipoprotein (oxLDL), and cholesterol can also trigger the activation of inflammasomes (Fig.1).

NLRC4 contains a CARD domain, therefore can directly recruit pro-caspase-1.^26^ The NLRC4 inflammasome mainly responds to bacterial flagellin and components of the bacterial type III secretory system (T3SS). Certain NLRC4-mediated functions may not require ASC, but do require interactions with the NLR family of apoptosis inhibitory proteins (NAIPs) which are receptors for bacterial protein ligands, and these interactions determine inflammasome specificity (Fig. 2).^37,38^ There are four NAIPs in mice: NAIP1 binds to the needle protein, NAIP2 detects rod proteins, and NAIP5/6 interact with flagellin.^39–42^ There is only one NAIP in humans, and it interact with the needle protein Cprl.^42^ In light of these findings, NLRC4 may be specific for the needle protein rather than flagellin in human infectious disease.

AIM2 is part of the inflammasome that can induce caspase-1 activation and subsequent cleavage of IL-1β and IL-18, contributing to the immune reaction against bacterial and viral infection. AIM2 inflammasomes directly bind to dsDNA (Fig. 3). Unlike NLRC4 inflammasomes, AIM2 does not contain CARD domains and thus needs to recruit ASC for activation.^43^ AIM2 not only senses bacteria and viruses derived cytosolic dsDNA, but also from damaged and mislocalized DNA molecules.^5,6,29,44,45^ Moreover, a minimum DNA length of 80 base pairs is required to activate AIM2.^9^ AIM2 is also involved in DNA vaccination,^46^ and may play a role in the development of systemic lupus erythematosus (SLE) due to its ability to recognize host DNA.^47^ However, more direct evidence is required to establish the role of AIM2 in the pathogenesis of SLE.

### Non-canonical inflammasomes

In mice, the non-canonical inflammasome is composed of caspase-11 and activated by cytosolic LPS (Fig. 4). Caspase-11 can activate both caspase-1 and caspase-3.^48^ Recently, caspase-11 was shown to indirectly activate NLRP3 inflammasome leading to the cleavage of pro-IL-1β or pro-IL-18.^30^ Importantly, caspase-11 senses intracellular LPS released by Gram-negative bacteria and induces pyroptosis and IL-1β secretion without involvement of the LPS receptor TLR4.^1,11,12^ Human cells do not possess the gene encoding caspase-11, but express caspase-4 and caspase-5, which have a similar function (Fig. 4).^31,49^ In response to LPS, caspase-4 can trigger the activation of caspase-1 without the need of second signals to activate the inflammasome.^49^ Along this line, caspase-11-deficient mice were resistant to a lethal dose of LPS injection.^30^ Further study of the non-canonical inflammasome will likely yield useful information to understand how human cells react to bacterial infection through PAMP-induced inflammation.

## Roles of inflammasomes in sterile inflammatory diseases

The inflammasome is a double-edged sword in various diseases, especially in sterile inflammatory diseases, and the outcomes can be either good or bad depending on the disease, and the genetic background.

### Inflammasomes and cardiovascular diseases (CVDs)

The role of the NLRP3 inflammasome in the development of CVDs and the treatment strategies targeting this key regulator of inflammation are discussed.

#### Inflammasomes and atherosclerosis

IL-1β was one of the first cytokines implicated in the pathogenesis of atherosclerosis. Various NLRP3 inflammasome stimuli that induce the production of mature IL-1β have been shown to contribute to the formation of atherosclerotic plaques.^50^ The NLRP3–IL-1β pathway has been identified as part of the innate immune memory system because it is a form of “trained immunity”, leading to a robust immune response to secondary infection.

**Fig. 5:**
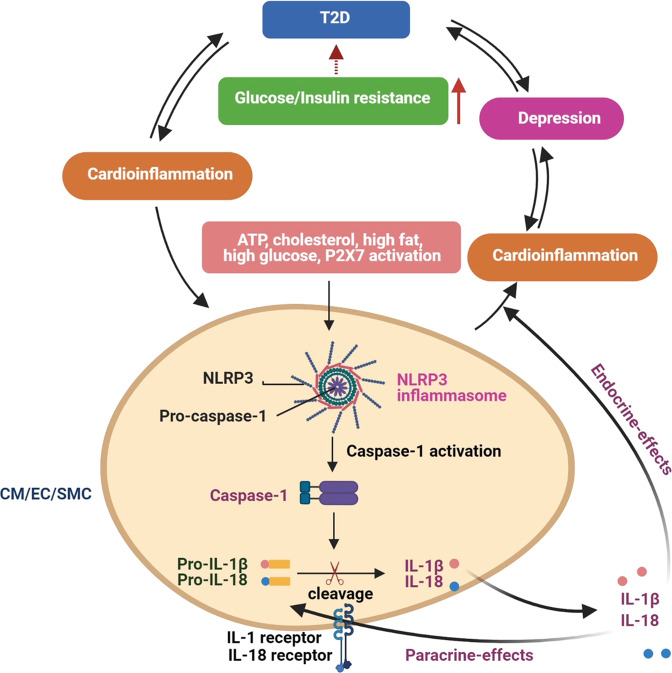
Inflammasome activation contributes to the development of diseases. The NLRP3 inflammasome is activated by DAMPs such as ATP, excess glucose, cholesterol, and high fat, leading to the activation of caspase-1 and production of active IL-1β and IL-18 in cardiomyocytes, endothelial cells, and smooth muscle cells. These risk factors are also involved in the development of depression and T2D. CM cardiomyocyte, EC endothelial cell, SMC smooth muscle cell, T2D type 2 diabetes

A recent study showed that the trained immunity can be induced not only by microbes but also by non-infectious agents such as a Western diet (WD). This study showed that WD-induced systemic inflammation returned to normal levels when mice were shifted back to a chow diet (CD).^51^ However, the inflammatory response of myeloid cells isolated from WD-fed mice to subsequent innate immune stimuli such as LPS remained elevated even after switching back to CD, suggesting that the myeloid cells retain memories of the WD stimuli. This trained immunity resembles the immune response to infectious agents or a vaccine. The investigators further showed that the trained immunity was due to reprogramming of myeloid progenitor cells at both transcriptional and epigenetic levels induced by the WD. The trained immunity did not occur in mice lacking NLRP3. Quantitative trait locus analysis in human monocytes confirmed that the NLRP3–IL-1β pathway is the mediator of trained immunity. These findings suggest that the harmful effects of a high-cholesterol diet are long-lasting and are mediated by the NLRP3–IL-1β pathway (Fig. 5).

The release of IL-1 induces the secretion of IL-6, which in turn promotes the synthesis of fibrinogen and plasminogen activator inhibitor to augment thrombosis and inhibit fibrinolysis, leading to the accumulation of thrombi in arteries.^52^ The critical role of IL-1 in the development of atherosclerosis highlights this cytokine as a potential target for intervention.^53,54^ Based on these findings, several clinical trials targeting the IL-1 signaling pathway have shown promising results (Table 1). A double-blind trial of canakinumab, designed to assess the therapeutic effect of a monoclonal antibody against IL-1β, showed beneficial effect in preventing the recurrence of myocardial infarction. Anakinra (an antagonist of the IL-1 receptor that inhibits the action of both IL-1α and IL-1β) and an antibody against IL-1α also showed beneficial effects.^55,56^ Animal studies demonstrated that IL-1α and IL-1β are involved at different stages during the development of atherosclerosis: neutralization of IL-1α limits early atherogenesis in the aortic root,^57^ whereas IL-1β blockade decreased the area of established atheromata. Their data indicate that IL-1α is involved in the remodeling of arteries during early atherogenesis, while IL-1β contributes to the formation of advanced atheroma.^57^

**Table 1:** Clinical trials targeting the NLRP3 inflammasome signaling pathway

Drugs	Diseases	Mechanism	Phase	Trial identifier
Anakinra	Metastatic breast cancer	Antagonist to IL-1 receptor (blocking both IL-1α and IL-1β)	Phase 1	NCT01802970
Anakinra	Metastatic colorectal cancer	Antagonist to IL-1 receptor (blocking both IL-1α and IL-1β)	Phase 2	NCT02090101
Anakinra/dexamethasone acetate	Multiple myeloma and plasma cell neoplasm	Antagonist to IL-1 receptor (blocking both IL-1α and IL-1β)	Phase 2	NCT00635154
Anakinra/dexamethasone	Indolent plasma cell myeloma, plasma cell myeloma	Antagonist to IL-1 receptor (blocking both IL-1α and IL-1β)	Phase 1	NCT02492750
Anakinra	Heart failure with normal ejection fraction	Antagonist to IL-1 receptor (blocking both IL-1α and IL-1β)	Phase 2	NCT02173548
Anakinra	Heart failure	Antagonist to IL-1 receptor (blocking both IL-1α and IL-1β)	Phase 3	NCT01936909
Anakinra	Heart failure	Antagonist to IL-1 receptor (blocking both IL-1α and IL-1β)	Phase 3	NCT01936844
Anakinra	ST segment elevation, acute myocardial infarction	Antagonist to IL-1 receptor (blocking both IL-1α and IL-1β)	Phase 3	NCT00789724
Anakinra	Heart failure	Antagonist to IL-1 receptor (blocking both IL-1α and IL-1β)	Phase 2	NCT01300650
Anakinra	Heart failure	Antagonist to IL-1 receptor (blocking both IL-1α and IL-1β)	N/A	NCT01542502
Anakinra	Acute myocardial infarction, heart failure	Antagonist to IL-1 receptor (blocking both IL-1α and IL-1β)	Phase 2	NCT01175018
Canakinumab	Non-small-cell lung cancer	Antibody targeting IL-1β	Phase 3	NCT03626545
Canakinumab	Non-small-cell lung cancer	Antibody targeting IL-1β	Phase 3	NCT03631199
Canakinumab	HIV, cardiovascular disease	Antibody targeting IL-1β	Phase 2	NCT02272946
Canakinumab	Type 2 diabetes	Antibody targeting IL-1β	Phase 2	NCT01068860
Canakinumab	Type 2 diabetes	Antibody targeting IL-1β	Phase 2	NCT00605475
Memantine/Dopamine receptor-agonists	Neurodegenerative disease	NLRP3 inhibition by blocking P2X7 receptor	N/A	NCT03918616
MCC950	Head and neck squamous cell carcinoma	Targeting NLRP3	N/A	N/A
BOT-4-one	Lymphoma	Targeting NLRP3	N/A	N/A

A clinical study showed that IL-18 expression in human atherosclerotic plaques is much higher than that of the normal arteries.^58^ Importantly, higher IL-18 mRNA levels are found in unstable plaques than stable plaques. IL-18 and its receptor have been identified in endothelial cells, smooth muscle cell, and mononuclear phagocytes within human atheroma, and it is able to stimulate the expression of IFN-gamma in cultured smooth muscle cells and mononuclear phagocytes.^58–60^ MCC950, an inhibitor of the NLRP3 inflammasome, was shown to reduce atherosclerogenic lesions in WD-fed apoE^−/−^ mice.^61^ MCC950 also improved cardiac function in a pig myocardial infarction model.^62^ Thus, the inhibition of inflammasome NLRP3 signaling represents a novel interventional therapy to prevent the development of atherosclerosis.^61–63^

#### Inflammasomes and other cardiovascular dysfunctions

IL-1β is a crucial cytokine promoting the inflammatory reaction in heart failure. Tet2 mutation in blood cells can increase atherosclerotic plaque size due to elevated IL-1β signaling in mice, and heart failure can be prevented by treating the mice with a specific NLRP3 inflammasome inhibitor.^64,65^ Activation of the inflammasome is also associated with aging. Furman et al. showed that individuals over 85 years of age, and those with increased levels of IL-1β, had a higher mortality rate due to nucleotide metabolism dysfunction. Interestingly, caffeine was shown to inhibit the metabolite-induced IL-1β production.^66,67^

NLRP3 inflammasome also plays an important role in the development of atrial fibrillation (AF). One recent study showed that the activity of NLRP3 inflammasome is increased in atrial cardiomyocytes (CMs) from patients with AF.^68^ CM-specific knock-in mice expressing constitutively active NLRP3 (CM-KI) developed spontaneous premature atrial contractions, which were blunted by the NLRP3 inhibitor MCC950. The CM-KI mice exhibited larger atria, abnormal calcium release from the sarcoplasmic reticulum, and electrical remodeling, which were prevented by CM-specific NLRP3 knockdown. Finally, genetic inhibition of NLRP3 prevented spontaneous AF in CREM (cAMP responsive element modulator) transgenic mice.^68^ These data demonstrate that both the electrical and structural remodeling associated with AF can be inhibited by targeting the NLRP3 inflammasome. Thus, NLRP3 inflammasome is a new therapeutic target for AF.

The NLRP3 inflammasome has been implicated in the development of pressure overload-induced cardiomyopathy. Suetomi et al. demonstrated that the activity of NLRP3 and caspase-1 is increased in the CM but not in the non-CM fraction containing fibroblasts, endothelial cells, and immune cells in response to transverse aortic constriction (TAC). These results indicate that the CM is the original site of NLRP3 inflammasome activation. Using CM-specific Ca^2+^-calmodulin-dependent protein kinase II (CaMKIIδ)-knockout (CKO) mice, they further showed that the increased levels of IL-1β, IL-18, and caspase-1 activity induced by TAC were attenuated in the CKO mice, suggesting that NLRP3 inflammasome activation in CMs is mediated through the CaMKIIδ signaling pathway. Mice treated with a CaMKII inhibitor in the first one or 2 weeks after TAC showed decreased fibrosis and ventricular dilation, but inhibition of CaMKIIδ after two weeks did not, suggesting that early intervention is required to prevent the development of cardiac remodeling and subsequent heart failure.^69^

In summary, the NLRP3 inflammasome plays a critical role in the development of CVDs, and NLRP3, caspase-1, and IL-1β can be exploited as new therapeutic targets. Some existing CVD medications have been shown to act via the NLRP3 inflammasome (Table 1), and further clinical assessment of these therapies is needed.

### Inflammasomes and depression

Depression is a common and serious mood disorder that affects 264 million people worldwide. Unlike short-lived mood-swings in response to stressful situations in everyday life, the symptoms of depression persist for a long time. Depression not only affects daily life, it can also lead to suicide. Stress is an important risk factor for depression. It is known that stress can induce inflammation, and depressive symptoms can be curbed by anti-inflammatory agents. The NLRP3 inflammasome functions as a bridge that links stress to immune activation.^70^

The role of the NLRP3 inflammasome in the pathogenesis of depression and mood disorders has been demonstrated in both animal models and patients.^70–75^ Peripheral diseases can activate NLRP3 inflammasome in the brain, leading to depression in rats.^71^ The expression of both NLRP3 and caspase-1 was shown to be increased in peripheral mononuclear cells from patients with major depressive disorder.^73^ Of note, depression is also associated with CVDs. High fat, cholesterol crystals, extracellular ATP, and excess glucose are risk factors that contribute to the development of CVDs by activating NLRP3 inflammasome, and these factors are also involved in the development of depression (Fig. 5).^76^ Dysregulated autophagy and mitophagy have been implicated in inflammasome activation and the development of both depression and CVDs.^77–80^ Several specific NLRP3 inflammasome inhibitors are being investigated for the treatment of both depression and CVDs. We anticipate that some of the inhibitors will prove useful in treating both diseases.

The NLRP3 inflammasome is involved in the development of diabetes, which is characterized by chronic elevation of blood glucose. Diabetes is due to either the failure of pancreatic β cells to make enough insulin (type 1) or the cells that express insulin receptors fail to respond to insulin (type 2; type 2 diabetes (T2D)). Depression and T2D may be mutually causal. People with depression tend to eat unhealthy food and have sedentary lifestyles that are risk factors for diabetes and CVDs. Conversely, diabetes can cause mood changes, anxiety, and frustration, which lead to depression.^81^

Two studies shed new light on the mechanisms underlying NLRP3 inflammasome activation. Extracellular ATP and neutrophils are abundant in the early stages of inflammation. ATP—induced activation of NLRP3 inflammasome and secretion of IL-1β is mediated by ionotrophic P2X_7_ receptors (P2X_7_R).^82^ IL-1β interfere with insulin signaling in depressed patients, leading to insulin resistance and T2D. Moreover, increased levels of IL-1β can trigger inflammation of the central nervous system, leading to reduced concentrations of serotonin (Fig. 5).^83^ Because P2X_7_R -mediated NLRP3 inflammasome activation is implicated in the development of both diabetes and depression, we expect that novel interventions targeting the P2X_7_–NLRP3–IL-1β pathway will reduce insulin resistance, leading to potential treatments for both depression and diabetes.

### Inflammasomes and neurodegenerative diseases

Alzheimer’s disease (AD) is a neurodegenerative disorder caused by the accumulation of misfolded Aβ peptide in the brain. Recent studies suggest that the deposition of Aβ peptide causes chronic inflammation.^84^ It is now clear that Aβ-induced activation of NLRP3 inflammasome leads to the synthesis of neurotoxic factors in microglia, which ultimately cause the development of AD.^23^ This study further showed that NLRP3 inflammasome activation is caused by the action of cathepsin B released from the lysosome rather than the direct actions of Aβ.^23^ These findings were confirmed by a separate study showing that cathepsin B inhibitors improve the memory deficit in transgenic AD mice.^85^ Subsequently, it was shown that the disruption of lysosome and release of cathepsin B are caused by CD36 which converts soluble ligands into insoluble fibrils.^86^ The connection between the NLRP3 inflammasome and the pathogenesis of AD was further confirmed using the gene-knockout approach in mice. NLRP3^−/−^ or caspase-1^−/−^ mice carrying AD mutations did not show loss of spatial memory.^87^ Furthermore, in APP/PS1/NLRP3^−/−^ mice (transgenic mice carrying an AD mutation crossed with NLRP3^−^^/−^ mice), caspase-1 cleavage was not seen and the brain IL-1β level was similar to wild-type mice. Importantly, qPCR analysis revealed increased mRNA levels of FIZZ1, arginase-1, and IL-4, suggesting that the microglial cells from APP/PS1/NLRP3^−/−^ mice are skewed to an M2 phenotype leading to increased Aβ clearance and enhanced tissue remodeling.^87^ Clinical studies have demonstrated increased active caspase-1 level in the brains of AD patients. Therefore, both animal and clinical studies (Table 1) revealed an important role for NLRP3 inflammasome in the pathogenesis of AD and strategies targeting the NLRP3–caspase-1 pathway are being investigated to find a cure for AD.^88–96^

Parkinson’s disease (PD) is a neurodegenerative disorder affecting dopaminergic motor neurons in the midbrain. The cause of the death of these neurons remains poorly understood, but is thought to be due to the abnormal uptake of aggregated α-synuclein (αSyn),^97^ which forms insoluble fibrils.^98^ Intracellular αSyn can also be transferred into extracellular fluid through exocytosis,^99^ which is a mechanism for spreading the misfolded αSyn to surrounding tissues, and the extracellular αSyn aggregates induce microglia activation and the secretion of IL-1β from astrocytes.^99,100^ Injection of an adenoviral vector expressing IL-1β into substantia nigra has been shown to trigger dopaminergic neuron death accompanied by microglia activation, infiltration of inflammatory cells, and akinesia.^101^ The fibrillary form of αSyn is able to activate caspase-1 and induce the production of mature IL-1β.^102^ The production of IL-1β is dependent on inflammasomes, which are activated by the phagocytosis of fibrillar αSyn, which in turn induces the production of reactive oxygen species (ROS) and excessive release of cathepsin B into the cytosol.^102^ NLRP3^−/−^ mice are resistant to neurotoxin MPTP-induced death of dopaminergic neurons and display reduced serum levels of IL-1β and IL-18 compared to the control mice.^103^ Dopamine prevents NLRP3 inflammasome activation by releasing the secondary messenger cAMP, which promotes the ubiquitination and degradation of NLRP3 through the E3 ubiquitin ligase MARCH7.^103^ Furthermore, mice lacking dopamine D1 receptors are more vulnerable to MPTP-induced neuroinflammation.^103^ These studies suggest that the dopamine signaling pathway and the NLRP3 inflammasome are mutually regulated, and PD patients may benefit from inhibition of the inflammatory process in the neurons.^104–108^

### Inflammasomes and insulin resistance

There are >500 million cases of T2D globally and this number is projected to increase significantly in the coming years. T2D is a chronic disease characterized by hyperglycemia due to insulin resistance.

The role of chronic inflammation in the development of T2D has been well documented,^109–111^ and the NLRP3 inflammasome was shown to mediate obesity-induced inflammation and insulin resistance.^112,113^ A number of molecules such as high glucose, islet amyloid polypeptide, saturated fatty acids, and mitochondrial ROS are involved in activating the NLRP3 inflammasome and contribute to the pathogenesis of T2D.^114,115^ The inflammasome and caspase-1 regulate adipocyte differentiation and insulin signaling, and a caspase-1 inhibitor improves insulin sensitivity in obese mice.^25,116–118^ Importantly, elevated inflammasome activation and caspase-1 have been found in both adipose tissue and myeloid cells from patients with T2D^119,120^ and high concentrations of IL-1β and IL-18 in the circulation are associated with an increased risk of T2D.^121,122^

One of the complications associated with T2D is the development of CVDs (Fig. 5). It has been shown that inhibitors of sodium-glucose cotransporter 2 (SGLT2) reduce the risk of developing CVDs in patients with T2D, and the beneficial effect is mediated by inhibiting NLRP3 inflammasome activity.^123^ The investigators showed that treatment with an SGLT2 inhibitor attenuates the activation of NLRP3 inflammasome and IL-1β production in patients with T2D and high cardiovascular risk by increasing serum β-hydroxybutyrate.^123^ These results opened a new door for the treatment of T2D and its complications by targeting the SGLT2-NLRP3-IL-1β pathway.

### Inflammasomes and cancer

Chronic inflammation mediated by inflammasomes plays a central role in tumorigenesis by altering the microenvironment and leading to neoangiogenesis, the proliferation of tumor cells, and metastasis. However, under certain conditions, inflammasome signaling also inhibits tumor growth by maintaining intestinal barrier integrity.^124,125^

#### Inflammasomes and the tumor microenvironment

The tumor microenvironment includes blood vessels, immune cells, extracellular matrix, and fibroblasts. Cancer-associated fibroblasts (CAFs) play a critical role in tumor growth and metastasis by activating inflammatory pathways.^126^

A recent study showed that NLRP3 inflammasomes are upregulated in human breast CAFs. They demonstrated that CAF-derived NLRP3/IL-1β facilitates tumor growth by recruiting myeloid-derived suppressor cells (MDSCs), thus altering the tumor microenvironment toward an immune-suppressive milieu.^126^ They further showed that tumor growth is delayed when NLRP3 or IL-1β is ablated. Activation of the inflammasome also contributes to resistance to chemotherapy. One study showed that although gemcitabine and 5-fluorouracil impede tumor-induced immunosuppression by killing MDSCs, they also activate NLRP3 inflammasomes in these cells, leading to IL-1β release that accelerates tumor growth.^127^ In contrast, other studies have shown that depletion of CAFs accelerates pancreatic cancer by recruiting the immunosuppressive FoxP3^+^ Treg cells.^128^ Others have shown that NLRP3 is required for suppression of metastasis of colorectal cancer (CRC) in the liver by promoting natural killer cell tumoricidal activity.^129^ In light of these conflicting reports, the NLRP3/IL-1β pathway can be either friend or foe in tumorigenesis, depending on the tumor type and context.^128–136^

#### Inflammasomes and metastasis

Tumor metastasis is a multi-step process in which both the primary tumor and the local microenvironment of the metastatic site play roles in tumor cell dissemination, metastatic colonization, and the formation of new tumors in specific distant organs.^137^ The metastatic sites of target organs form an immune-suppressive environment and a pre-metastatic niche to prepare for the arrival of metastatic cells.^138^ Circulating tumor cells must be able to adhere to capillary endothelia for metastasis to occur. It has been shown that IL-1β promotes the metastasis of melanoma cells to liver by up-regulating vascular cell adhesion molecule.^139^ Tumor growth and metastasis requires a sufficient blood supply. IL-1β is a necessary cytokine to induce tumor angiogenesis, and IL-1β^−/−^ mice exhibit impaired blood vessel growth and tumor development.^140^ The role of IL-1β in tumor metastasis is further supported by data showing that an IL-1R antagonist suppresses metastases of melanoma and improves survival of mice.^134^ The effects of inflammasome activation on tumor growth and metastasis may be mediated by different cytokines, depending on the cancer type and metastatic site. In the CRC model of intrasplenic injection of colon cancer cells, inflammasome-mediated IL-18 production is responsible for the suppression of tumor growth in the liver.^129^ However, another study showed that NLRP3-mediated IL-1β secretion promotes the dissemination of colon cancer cells.^141^ In a carcinogen-induced tumor model, NLRP3 promotes metastasis by suppressing natural killer cell activity.^142^

#### Inflammasomes and tumor-suppression

The tumor-suppressive role of the NLRP3,^143,144^ AIM2,^145,146^ and NLRC4^147,148^ inflammasomes is well documented in colon cancer. The NLRP3 inflammasome deficient mice are more susceptible to colitis-induced colorectal cancer due to reduced production of IL-18, and subsequent inactivation of tumor suppressor STAT1. The expression of AIM2 is reduced in cancer cells and AIM2 inhibits tumor growth by interacting with DNA-dependent protein kinase, leading to inactivation of AKT^145,149^ and inhibition of the proliferation of colonic stem cells.^146,149^ Caspase-1-deficient mice shows enhanced tumor growth in inflammation-induced colorectal cancer model and the tumor-suppressive function of caspase-1 is mediated by NLRC4.^147,148^ Of note, ASC is a tumor suppressor in keratinocytes, but a tumor-promoter in myeloid cells.^150^ These findings suggest that the roles of inflammasomes in tumor-suppression are cell type dependent.

Taken together, the activation of inflammasomes can either enhance or inhibit tumor progression and metastasis depending on tumor type and context. Further studies are required to understand how inflammasomes are regulated in each tumor type and to determine their contribution to tumor development.

### Gene polymorphisms of inflammasomes and disease

Gene polymorphisms of inflammasomes and related cytokines are associated with the pathogenesis and prognosis of various diseases such as cancer, CVDs, and T2D.

#### Gene polymorphisms of inflammasomes in tumors

Gene polymorphisms of inflammasomes are associated with an increased risk of developing cancers. Several single-nucleotide polymorphisms (SNPs) in the NLRP3 region is associated with increased production of IL-1β that contribute to susceptibility to Crohn’s disease,^151,152^ which is a risk factor for the development of CRC. A heterozygous NLRP3 (Q705K) variant is associated with poor survival in patients with invasive CRC.^153^ The AIM2 gene contains a coding region frame-shift mutation caused by microsatellite instability in CRC.^154,155^ In addition, regression analysis has shown that AIM2 can promote the development of endometrial cancer.^156^

The polymorphisms of genes involved in the NLRP3 inflammasome pathway may be associated with the prognosis of chronic myeloid leukemia (CML). One study investigated the association of genetic polymorphisms of NLRP3, IL-1β, IL-18, and CARD8 with CML. They showed that the AT genotype of CARD8 (rs2043211) was more frequent in high- and intermediate-risk CML. CARD8 is an NLRP3 binding partner that inhibits tumor development by suppressing NF-κB. The rs2043211 variant is translated to a truncated CARD8 protein that is unable to inhibit NF-κB. The gene polymorphisms of both IL-18 (rs1946518) and IL-1β (rs16944) are associated with CML. Therefore, polymorphisms of the NLRP3 inflammasome may provide useful information for predicting the clinical outcome in response to treatment.^157^

#### Gene polymorphisms of inflammasomes in other diseases

Gene polymorphisms of inflammasome components have also been implicated in the development of CVDs. One study evaluated the impact of NLRP3 (exon 3) gene polymorphisms and serum NLRP3 levels on the risk of developing myocardial infarction (MI) in 69 patients and 53 controls. They identified five SNPs that were associated with a high incidence of MI. Therefore, genetic polymorphisms of NLRP3 may be used as indicators for predicting the risk of developing MI.^158^

The association between the genotypes of SNPs in the NLRP3 downstream regulatory region and NLRP3 mRNA expression was investigated in the Swedish First-ever myocardial Infarction study consisting of DNA from 555 MI patients and 1016 healthy individuals. Expression of NLRP3 inflammasome components was significantly higher in carotid artery plaques than in normal arteries. The rs6672995 and rs10733113 variants were associated with NLRP3 mRNA levels in blood cells. These results suggest that genetic variants of the NLRP3 inflammasome are involved in the development of atherosclerosis.^159^

Chronic inflammation driven by excess body fat is a common feature of T2D. A randomized dietary intervention with Mediterranean (Med) and low-fat diets was conducted to determine whether insulin sensitivity is associated with diet and genetic factors. After 3 years of intervention, SNPs located at the NLRP3 gene were determined in both diabetic and non-diabetic patients. The results showed that insulin sensitivity was improved in non-diabetic patients carrying the SNPs rs4612666 and rs10733113, whereas no effects were found in the diabetic patients. The results suggest that gene polymorphisms of NLRP3 are associated with insulin sensitivity in non-diabetic patients.^160^

Taken together, SNP analysis may provide useful information for understanding how individual components of the inflammasome contribute to the pathogenesis and prognosis of diseases. Future studies investigating how SNPs contribute to disease phenotypes and treatment outcomes will delineate the molecular mechanisms whereby inflammasomes influence the development and progression of disease.

## Therapeutic strategies targeting the inflammasome signaling pathway

Several recent studies targeting NLRP3 inflammasome, including IL-1, have shown promising beneficial results.^56,124,161^

**Fig. 6:**
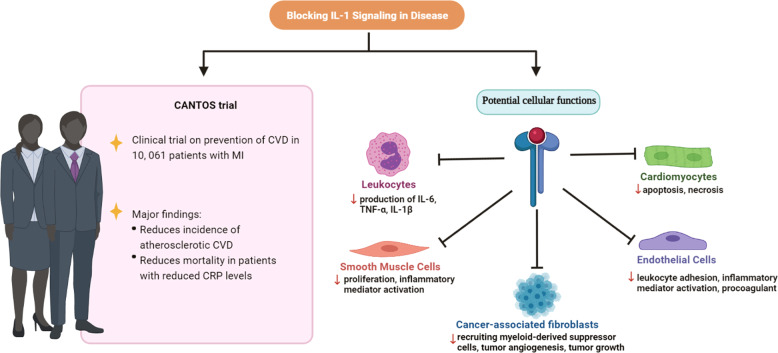
Mechanisms underlying the beneficial effect of IL-1 blockade in treating cardiovascular diseases and cancer. The CANTOS trial showed a reduced incidence of CVD in patients treated with canakinumab, a monoclonal antibody blocking IL-1β. The potential mechanisms underlying the beneficial effects of IL-1 targeting strategies include inhibition of cardiomyocyte apoptosis and necrosis, reduction of inflammatory mediator activation in endothelial cells, prevention of recruitment of tumor suppressor cells, and inhibition of tumor angiogenesis and tumor growth. CRP C-reactive protein, CVD cardiovascular disease

The Canakinumab Antiinflammatory Thrombosis Outcome Study (CANTOS) demonstrated that inflammation has a central role in the pathogenesis of atherothrombosis.^162^ This randomized clinical trial recruited 10,061 patients who were treated with either Canakinumab, a monoclonal antibody blocking IL-1β, or a placebo. All patients had a previous bout of MI and increased levels of high-sensitivity C-reactive protein, the latter an indicator of ongoing inflammation. The primary end-point was a combination of non-fatal MI, non-fatal stroke, or cardiovascular death. The results showed that Canakinumab reduced the rate of recurrent cardiovascular events. Therapeutic approaches targeting IL-1α and IL-1 receptor also showed beneficial effects.^55,56^ The beneficial effects were achieved by inhibiting the release of cytokines from leukocytes and abrogating proliferation of smooth muscle cells. Likewise, apoptosis and necrosis of CMs were reduced and leukocyte adhesion to endothelial cells was diminished (Fig. 6).

Blockade of cytokines has shown promising results in delaying or preventing cancer progression. A phase II clinical trial investigated the effect of anakinra, an IL-1 receptor antagonist (IL-1Ra), in patients with indolent multiple myeloma showed that anakinra decreased the myeloma proliferation rate.^163,164^ IL-1Ra blocks the signaling pathway activated by both IL-1α and IL-1β. Whereas the production of IL-1β is mostly mediated by inflammasome, the release of IL-1α could be either inflammasome-dependent or -independent based on the activator.^165^ A phase III clinical trial has demonstrated that MABp1, a monoclonal antibody blocking IL-1α, improves the survival rate in patients with refractory CRC.^166^

Targeting the pro-inflammatory IL-1β pathway with canakinumab has been shown to inhibit the development and progression of lung cancer.^167^ The results from a phase II clinical trial showed that co-administration of anakinra with 5-fluorouracil and bevacizumab improves the median survival rate in patients with metastatic CRC.^168^ These promising results demonstrate that the anti-inflammasome strategy is an effective approach for cancer treatment and its efficacy can be improved with a combination therapy. The therapeutic effect of canakinumab is mediated by blocking the IL-1 signaling in CAFs, leading to reduced infiltration of tumor suppressor cells and inhibition of tumor angiogenesis as well as tumor growth (Fig. 6).

Although the anti-IL-1β strategy is successful in preventing recurrent cardiovascular events, it is associated with a higher incidence of fatal infections as shown in the CANTOS trial. This side-effect is likely due to suppression of the immune system. To overcome this problem, Li and colleagues have fabricated platelet microparticles (PMs) armed with the anti-IL-1β antibodies that specifically block the IL-1β signaling pathway in the injured heart. Administration of platelets carrying the anti-IL-1β PMs prevented adverse cardiac remodeling after MI by inhibiting caspase-3 activity and protecting the CMs from apoptosis. Therefore, strategies designed to target specific cells or organs are expected to make IL-1β antibody safer and more efficient.^169^ Tissue-specific blocking of inflammasome signaling may facilitate the development of more efficient therapeutic strategies.

## Conclusions and perspectives

**Fig. 7:**
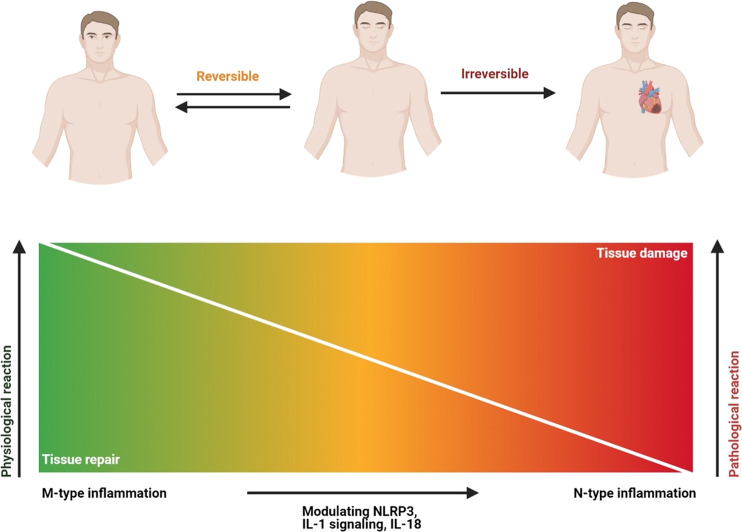
Beneficial effects of acute inflammation and deleterious effects of chronic inflammation. Acute inflammation (M-type) is a beneficial process to destroy pathogens and initiate tissue repair. However, chronic inflammation (N-type) is a pathological condition that lasts for a long time, leading to tissue damage. Both types of inflammation can be modulated by the inflammasome

In this review, we summarized the link between inflammasomes and sterile inflammatory diseases, including CVDs, depression, neurodegenerative diseases, diabetes, and cancer. Stress, smoking, poor diet, and a sedentary lifestyle are common risk factors for diabetes, CVDs, and depression. These risk factors activate inflammasomes, which in turn leads to chronic inflammation that contributes to insulin resistance, atherosclerosis, and mood change. However, in the case of cancer, inflammasomes can be either friend or foe depending on the cell type and context. Activation of inflammasomes in acute diseases helps to remove dead cells and initiate tissue repair. However, persistent activation of inflammasomes in chronic diseases is detrimental because it damage tissues (Fig. 7). In order to explore the potential of inflammasomes as therapeutic targets, a greater understanding of the signaling molecules mediating the beneficial and detrimental effects of their activation is needed.

As strategies targeting IL-1 enter clinical trials, we face new challenges as well as opportunities. The CANTOS trials confirmed that inflammasome activation is the cause rather than a consequence of atherothrombosis. This is an important step toward the clinical application of therapeutic strategies targeting the inflammasome signaling pathway. However, the trial also revealed an increased incidence of fatal infection due to off-target effects, which must be properly addressed before canakinumab can be routinely used to prevent cardiovascular events. To solve this problem, Li et al.^169^ introduced PMs as carriers to deliver the IL-1β antibody to the injured heart and demonstrated promising results. This approach not only reduces the risk of side-effects but also improves therapeutic efficiency.

In addition to IL-1 blockade, inhibition of NLRP3 could also be beneficial in certain diseases. Along this line, MCC950, a specific and potent inhibitor of NLRP3, has been shown to reduce the development of atherosclerogenic lesions in WD-fed apoE^−^^/−^ mice.^61^ MCC950 reduces infarct size in a pig myocardial infarction model,^62^ protects against diabetes-associated atherosclerosis in mice,^170^ and improves spatial memory in SAMP8 mice, an animal model of AD.^171^ In addition, NLRP3 silencing in apoE^−/−^ mice inhibits the induction of IL-1β and IL-18 and prevents the progression of plaques.^172^ These studies point to a potential new therapeutic avenue for treating sterile inflammatory diseases.

In summary, NLRP3 acts as a nodal factor which can be beneficial after either activation or inhibition. Aberrant NLRP3 activation is implicated in chronic inflammation that leads to the development of cancer, aging, and degenerative diseases. IL-1 blockers such as canakinumab, anakinra, and rilonacept are only effective in treating certain NLRP3-mediated diseases. The development of more effective treatment relies on a thorough understanding of the signaling pathways triggering the activation of the inflammasome (Box 2). In addition, a better understanding the mechanisms underlying the regulation of non-canonical inflammasome responses and the interaction between the non-canonical inflammasome and the canonical inflammasome may lead to the development of effective therapies for a broader range of pathological conditions.

Box 2 Key points • *Double-edged sword*: The studies show that the role of inflammasomes in the pathogenesis of diseases is bidirectional, and the outcomes can be either good or bad depending on the types and stages of disease, as well as the genetic background.

• *Inflammasome memory*: In this process, the NLRP3–IL-1β pathway is an important mediator of “trained immunity”, which is defined as the ability of the innate immune system to undergo reprogramming and form an immune memory that provides long-lasting protection against pathogens.

• *Two-shot activation*: Activation of the inflammasome is a two-step process that requires two shots. The first shot is PAMP and DAMP-induced upregulation of NLRP3 and pro-IL-1β expression, which occurs at both the transcriptional and post-translational levels. The second shot is oligomerization of the inflammasome in the cytosol.

• *M-type or N-type inflammation model*: M-type inflammation is a process to remove a pathogen by the inflammatory response, which then returns to normal. N-type inflammation is an over-reaction of the inflammatory response that becomes chronic inflammation leading to tissue damage.

• *Targeting the NLRP3/caspase-1 pathway*: Although clinical trials have shown promising results in treating various diseases by inhibiting downstream effector molecules such as IL-1β and IL-18, strategies blocking the NLRP3/caspase-1 pathway represent a better approach, because caspase-1 blockade can inhibit global inflammatory responses regulated by both inflammasome-dependent IL-1β/IL-18 and pyroptosis. In addition, inhibition of a specific inflammasome such as NLRP3 by MCC950 can block its pathological effects without affecting the beneficial effects of other inflammasomes. Thus, the development of highly specific NLRP3/caspase-1 inhibitors will pave the way to the development of new therapeutic strategies for clinical application.
